# Altered GABA Signaling in Early Life Epilepsies

**DOI:** 10.1155/2011/527605

**Published:** 2011-07-31

**Authors:** Stephen W. Briggs, Aristea S. Galanopoulou

**Affiliations:** Saul R. Korey Department of Neurology, Dominick P. Purpura Department of Neuroscience, Albert Einstein College of Medicine, 1410 Pelham Parkway South, Kennedy Center Rm 306, Bronx, NY 10461, USA

## Abstract

The incidence of seizures is particularly high in the early ages of life. The immaturity of inhibitory systems, such as GABA, during normal brain development and its further dysregulation under pathological conditions that predispose to seizures have been speculated to play a major role in facilitating seizures. Seizures can further impair or disrupt GABA_A_ signaling by reshuffling the subunit composition of its receptors or causing aberrant reappearance of depolarizing or hyperpolarizing GABA_A_ receptor currents. Such effects may not result in epileptogenesis as frequently as they do in adults. Given the central role of GABA_A_ signaling in brain function and development, perturbation of its physiological role may interfere with neuronal morphology, differentiation, and connectivity, manifesting as cognitive or neurodevelopmental deficits. The current GABAergic antiepileptic drugs, while often effective for adults, are not always capable of stopping seizures and preventing their sequelae in neonates. Recent studies have explored the therapeutic potential of chloride cotransporter inhibitors, such as bumetanide, as adjunctive therapies of neonatal seizures. However, more needs to be known so as to develop therapies capable of stopping seizures while preserving the age- and sex-appropriate development of the brain.

## 1. Introduction

Epilepsy is a disease of recurrent seizures: that is, unprovoked episodes of aberrant synchronous excitation of brain regions that disrupt normal functioning [1, 2]. Epileptic seizures are thought to reflect a failure in the ability to maintain the balance between excitation and inhibition. The mechanisms underlying seizures are complex and not uniform across the numerous seizure types that exist [1]. Furthermore, our ability to study these mechanisms is often limited by the tools we can use: we can only see as far and as much as those tools allow. Consequently, many of the hypotheses describing the pathogenesis of seizures are biased by the dominant ictal phenomena, unbalanced excitation-inhibition and aberrant neuronal synchronization, which may not necessarily be the actual ictogenic mechanisms. Neurotransmitters involved in neuronal inhibition, such as GABA, have attracted the major focus of research aiming to decipher mechanisms involved in ictogenesis. Under certain conditions, and definitely not in the majority of cases, seizures may lead to epilepsy or neurodevelopmental deficits. The early periods of life, when brain development is still incomplete, susceptibility to seizures is increased [3, 4]. However, a combination of biological factors (genetic, age-related processes, epigenetic or environmental factors) protect neurons from seizure-induced injury, epileptogenesis, or mortality to a greater extent than the adult brain is protected [5]. It is increasingly recognized that seizures may leave their imprint on the developing brain by altering the way that neurons differentiate, connect, and communicate to each other, even if, in many cases, such changes may be ultimately compensated for. As extensively outlined in the reviews included within this special issue, GABA plays a central role in controlling neuronal development and communications. A major focus of research has therefore been thrown into efforts to elucidate its role not only in ictogenesis but also in the pathogenesis of the sequelae of early life seizures, whether this may be epilepsy, cognitive, or behavioral deficits [6]. 

There are three types of GABA receptors reported in the literature: GABA_A_, GABA_B_, and GABA_C_, the latter classified more recently along with GABA_A_ receptors, due to their functional similarities. Both GABA_A_ and GABA_C_ receptors are ligand-gated ionotropic channels that allow primarily chloride but also bicarbonate to cross their pore in response to GABA binding. GABA_B_ is a metabotropic receptor that signals through cascades that modify potassium and calcium current (reviewed in [7]), direct migration [8], and control gene transcription [9, 10]. In this review, we will focus primarily on GABA_A_ receptors.

GABA_A_ receptors are pentameric channels usually comprised of 2 *α* and 2 *β* subunits, whereas the fifth is either a *γ* or a *δ* subunit. Less frequently, *ε*, *θ*, or *π* subunits are present [11–13]. There are 16 known mammalian GABA_A_ receptor subunits (*α1*−*α6*, *β1*−*β3*, *γ1*−*γ3*, *δ*, *ε*, *θ*, *π*), which contribute towards the different pharmacokinetic, subcellular localization or affinity properties of each GABA_A_ receptor complex. The presence of a *ρ* subunit defines the GABA_C_ receptors. Unlike GABA_A_ receptors, GABA_C_ are insensitive to bicuculline. The expression of GABA_A_ receptor subunits changes with development and as a result the responsiveness of immature and adult neurons to GABA_A_ ergic modulators are significantly different.

**Figure 1:**
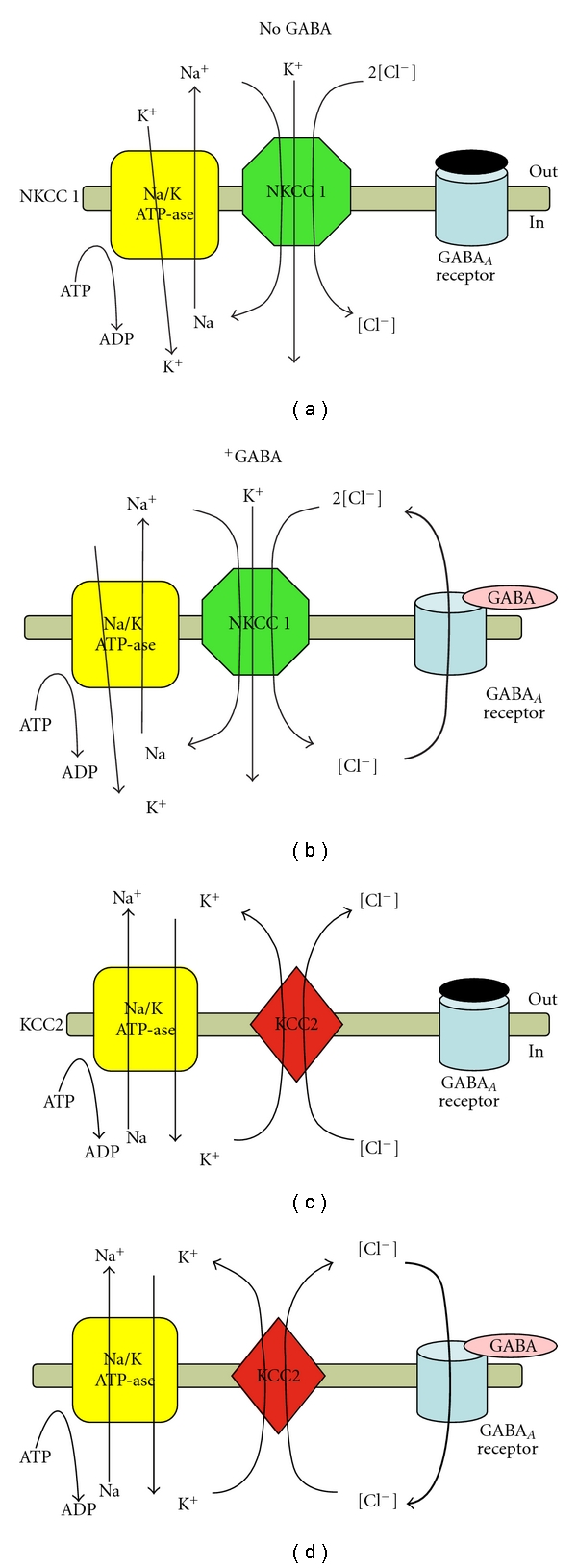
CCCs control GABA_A_ receptor-mediated inhibition. Panels (a) and (b) show the effects of NKCC1 activity in the absence (panel (a)) or presence (panel (b)) of GABA. NKCC1 mediates the electroneutral cotransport of Na^+^, K^+^, and 2 Cl−, increasing the intracellular Cl^−^ concentration. As a result, upon binding of GABA upon the GABA_A_ receptor, the channel pore opens and Cl leaves the neuron, causing a depolarization. Panels c and d show the effects of NKCC1 activity on GABA_A_ receptor function in the absence (panel c) or presence (panel d) of GABA. KCC2 in contrast exports K^+^ and Cl^−^ reducing intracellular Cl^−^. Activation of GABA_A_ receptors therefore results into influx of Cl and hyperpolarizing current. Their function is dependent upon the gradients of Na^+^ and K^+^, which are controlled by various factors, including background conductances, membrane voltage, and by the Na^+^/K^+^ ATPase.

The classical inhibitory GABA_A_ signaling, as occurs in most adult neurons, is due to chloride influx through the channel pore, which hyperpolarizes the cells. This is achieved because the intracellular chloride concentration is maintained at a low level, allowing chloride to flow in along its electrochemical gradient, when GABA_A_ receptors open (Figure 1). Multiple studies over the last few decades have confirmed that this electrochemical chloride gradient is developmentally regulated by changes in the expression of cation-chloride cotransporters (CCCs). CCCs are the electroneutral ion symporters that establish the chloride gradient between cells and their extracellular environment. There are 3 CCC classes. The chloride importing CCCs are either the sodium/potassium/chloride cotransporters (NKCCs), with known representatives the NKCC1 and NKCC2, or the sodium chloride cotransporters (NCCs). Chloride exporters are the potassium/chloride cotransporters (KCCs), with 4 known isoforms: KCC1-4 (reviewed in [11, 12, 14, 15]) (Figure 1). Immature neurons express predominantly chloride-importers, such as NKCC1 [16], which generate high intracellular Cl^−^ levels. This forces the open GABA_A_ receptors to permit Cl^−^ efflux through their channel pore, giving rise to depolarizing GABA_A_ responses [16–18]. During developmental maturation, the expression of chloride-extruding CCCs, like the potassium/chloride cotransporter 2 (KCC2), dominates over NKCCs [19–22], decreasing the intracellular chloride concentration [23]. As a result, when GABA opens GABA_A_ receptors the ensuing influx of chloride results in hyperpolarizing currents [19] (Figure 1). However, cell type, sex, and species/strain differences occur in the timing of this developmental shift. KCC1, KCC3 and KCC4 are widely expressed, but KCC2 is specific to neurons. This makes KCC2 particularly interesting for the pathogenesis and therapy of neural diseases. NKCC2 expression is specific to the kidney, leaving NKCC1 as the most relevant chloride-importing cotransporter for the brain, though it is expressed ubiquitously. Bicarbonate, generated by carbonic anhydrase, is another negatively charged ion that can permeate the GABA_A_ receptor, generating a depolarizing response [12, 24, 25]. The cytosolic carbonic anhydrase VII (CAVII) increases around postnatal day 12 (PN12) in the rat hippocampus [26], rendering bicarbonate-mediated GABA_A_  depolarizations more prominent [25].

There is considerable evidence that alterations in GABA signaling can cause seizures, as well as that seizures can change GABAergic signaling. In this review, we will discuss the bidirectional relationship of seizures to GABA_A_ signaling at the level of the neurons, GABA_A_ receptors, and the ionic symporters that control chloride homeostasis and the efficiency of GABA_A_ receptor mediated inhibition.

## 2. Correspondence of Developmental Stages between Rodents and Humans

To facilitate the translation of the experimental data into humans, it is worth reminding that the accepted correspondence of developmental stages between rodents and humans considers that the first week of life in rodents is equivalent to a premature newborn human, whereas the time of birth in rodents is considered to correspond to PN8-10. The rodent infantile stage is thought to extend till PN21, the onset of puberty is at PN32-35 in rodents, whereas PN60 rodents are considered young adults. However, it is important to emphasize that this is a very oversimplified translation, based mostly on correspondence of protein and DNA content in the brain. Each developmental process occurs at different tempos and is not always in synchrony with the above sequence of events. For example, by the end of the first postnatal week, rats are able to walk away from the nest, quite unlike the human newborns who cannot yet ambulate [27]. Direct demonstration of the time of shift of GABA_A_ receptor responses to hyperpolarizing has not been demonstrated in humans, though it has been suggested to occur before or soon after birth, based on the developmental patterns of the relative expression of NKCC1 and KCC2 [21, 28].

## 3. The Immaturity of GABA_A_ergic Systems as an Age and Sex-Specific Risk Factor for Early Life Seizures

Seizures are more common in the early periods of life and especially in males [3, 4]. The immaturity of GABAergic inhibitory systems has been implicated in the heightened susceptibility of neonates to seizures and may also underlie the increased vulnerability of males, in whom the maturation of these systems is delayed compared to females. GABA is depolarizing in the neonatal life and it stays depolarizing for longer developmental periods in the male brain than in females [17, 29–33]. Paradoxical exacerbation of seizures by GABA-acting drugs has been reported in newborns, especially in low weight premature babies [34]. GABA-acting drugs, such as benzodiazepines and barbiturates, however, still remain the mainstay of treatments for neonatal seizures, even if they may not always be as effective in newborn human babies as in older patients [21, 35–39]. This is thought to be due to shunting inhibition or inhibition via excitatory effects upon inhibitory interneurons [40]. The composition of GABA_A_ receptors is also different in newborns, with less *α*1 and more *α*2/3 subunits, rendering them less responsive to benzodiazepines [41, 42]. Furthermore, the subcortical GABAergic networks that control seizures, like the substantia nigra pars reticulata (SNR), have not fully developed [31, 42–46]. The excessive GABAergic stimulation of the SNR, as is thought to occur due to GABA release during seizures, has proconvulsant effects early in life and anticonvulsant in older animals and this switch occurs earlier in females [44, 45]. It is therefore important to investigate and clarify the exact molecular determinants that control GABA_A_ inhibition in the young brain so as to optimize the treatment of seizures.

## 4. Aberrant GABA_A_ Signaling Predisposes to Seizures

**Figure 2:**
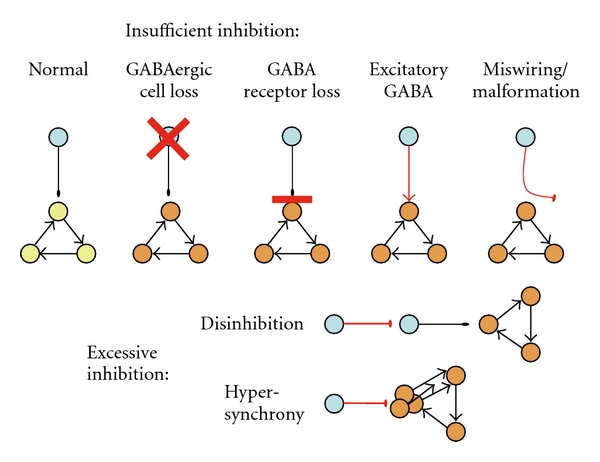
Schematic depiction of simple models through which dysregulation of GABA_A_ receptor-mediated inhibition can increase the activity of neuronal networks, potentially generating seizures. GABA inhibition can fail when GABA or GABA_A_ receptor expression is low, when GABA depolarizes neurons, or when miswiring and mistargeting of synapses occur. Excessive GABA inhibition may trigger seizures by disinhibiting target cells, or via excessive synchronization of the neurons in the epileptogenic focus. Please note that the effects of dysregulated GABA signaling in more complex neuronal networks, especially in the presence of abnormal circuitry or with specific pathologies, may differ. In such cases a combination of the above models may be applicable at different sites of the epileptogenic network rendering the pharmacological effect of a GABAergic agonist not completely predictable by a single model. Furthermore, shunting inhibition may explain situations where GABAergic drugs silence excessive excitatory network activity, in neurons with depolarizing GABAergic signaling.

Clinical and experimental evidences indicate that an initial perturbation of GABA_A_ signaling may facilitate seizures. A loss of inhibition could result in runaway excitatory circuits. Too much inhibition could also cause a seizure, either by disinhibiting epileptogenic networks or via promoting neuronal synchronization ([67] reviewed by [68]). Excessive inhibition has been implicated in autosomal dominant nocturnal frontal lobe epilepsy (ADNFLE) ( [69] reviewed in [70]) or absence seizures [71]. Moreover, as GABA_A_ signaling is critical for brain development and early synaptogenesis [72–74], a disorder of GABA_A_ signaling early in life may cause miswiring or malformations that predispose to seizures (Figure 2).

Many GABA-related mutations are known to cause early life epilepsy. These include loss of function mutations or deletions of GABA_A_ receptor subunit genes that reduce their expression, or the duration, amplitude or agonist sensitivity of GABA_A_ currents. GABA_A_ receptor subunit mutations have been implicated in childhood absence epilepsy (CAE) [50, 51, 75], autosomal dominant epilepsy with febrile seizures plus (ADEFS^+^) [76], and other epileptic syndromes (reviewed in Table 1 and [77, 78]). Conditional mutants indicate that the developmental period of exposure to insults that disrupt GABA_A_ signaling may be critical in ictogenesis and epileptogenesis. Chiu et al. proposed that loss of function mutations of the GABA_A_ receptor subunits may have developmental effects in addition to their direct electrophysiological consequences [79]. Using a conditionally expressed loss of function mutation of the *γ*2 GABA_A_ receptor subunit in mice, the investigators expressed the mutant allele for different periods of time. Mice that were induced to express the mutant allele for longer developmental periods displayed higher seizure susceptibility to pentylenetetrazole (PTZ), a drug that acts as a GABA_A_ receptor antagonist, compared to mice with late disruption of the *γ*2 subunit expression.

Glutamic acid decarboxylase (GAD) isoforms GAD65 and GAD67 synthesize GABA in the brain. Knockout mice for the pyridoxal-5′-phosphate inducible GAD65 isoform, that generates the GABA reserve pools, have lower seizure threshold to picrotoxin, a GABA_A_ receptor antagonist [61], or spontaneous seizures that can be precipitated by stress [60]. Although total GABA content in the brain may be normal or decreased in GAD65 knockout mice, depending upon the genetic substrate, it has been proposed that GAD65 loss of function may preferentially decrease the presynaptic reserve pool of GABA and decrease the tonic GABA inhibition, leading to increased seizure susceptibility [80–82]. Although no human GAD mutations have been found to consistently cause epilepsy [83], mutations in cofactors that are necessary for GAD65 function have been linked with early life seizures, as occurs in pyridoxine-dependency disorders [84, 85]. GAD65 or GAD67 loss sufficiently compensates for each other and does not appear to affect early brain development; albeit, cleft palate has been reported with GAD67 knockout mice [86]. Dual GAD65/67 knockout mice are not viable [87]. A small subset of patients manifests epilepsy secondary to an autoimmune response against GAD65/67, although these appear mostly in adults [88–91].

## 5. Disrupting CCC Function May Predispose to Seizures

Decreased expression or function of chloride extruders may change seizure susceptibility by not only diminishing the efficacy of GABA_A_ inhibition, and promoting cellular swelling and degeneration under hypotonic conditions, but also by exerting broader developmental effects. Human linkage studies or transgenic knockout animal studies document that, at least in certain cases, seizures and epilepsy may ensue. There is currently no known human mutation of KCC2 associated with epilepsy. This may rather reflect the indispensability of KCC2, as complete KCC2 knockout mice die postnatally from respiratory failure, due to the immaturity of the respiratory system [93]. KCC2 has two known isoforms, KCC2a and KCC2b, of which KCC2b is thought to contribute to the developmental shift to hyperpolarizing GABA_A_ receptor currents [106]. KCC2b-knockout mice demonstrate hyperexcitability at PN10 to PN16 (equivalent to human infantile age) [94] (Table 2). Although the expected intracellular accumulation of chloride and depolarizing shift of GABA_A_ responses could easily explain the hyperexcitability, application of the GABA_A_ receptor antagonist picrotoxin paradoxically retains its excitatory responses [94]. Similarly, a different hypomorphic mutation in KCC2 causes a lower PTZ threshold for induction of clonic seizures in mice, despite the absence of gross morphological changes [95]. Such observations are indicative of a residual inhibitory capacity of KCC2, either in the form of less potent hyperpolarizing GABA_A_ receptor currents or shunting inhibition [107]. However, the function of KCC2 is more complex, due to interactions with dendritic cytoskeletal proteins [108] or with other modulators of neuronal activity (i.e., increasing extracellular potassium) [109] which need to be further analyzed as to their ability to influence the phenotype of these mice.

Loss of function mutations in KCC3, which is expressed in many tissues, have been reported in patients with hereditary motor sensory neuropathy, some of whom have seizures as well as developmental deficits, like agenesis of the corpus callosum [100].

Altered CCCs may also affect brain development in a more subtle fashion, which could predispose a brain to epilepsy even if it does not directly cause seizures. From various fronts evidence emerges that shifts in the timing of emergence of hyperpolarizing signaling may have significant impact on neuronal and brain development and connectivity. Precocious appearance of hyperpolarizing GABA_A_ receptor signaling, either by KCC2 overexpression [72] or via loss of NKCC1 activity [110], disrupts cortical morphogenesis. Pharmacological inhibition of NKCC1 with bumetanide from embryonic day E15 to PN7 in otherwise normal mice disrupts cortical dendritic formation [74]. Abnormal cortical development and synaptic connectivity may predispose to seizures or cognitive impairment, which is both a predisposing factor and a common comorbidity of young patients with epilepsy [111].

## 6. Secondary Disruption of GABAergic Signaling in Risk Factors for Early Life Epilepsy

Conditions that predispose to epilepsy, genetic or acquired, may also create an imbalance in excitation/inhibition. Although their effects are not restricted to GABA_A_ signaling, in certain cases they may show a predilection to preferentially impair GABAergic inhibition.

Mutations of the aristaless-related and X-linked homeobox gene ARX have attracted a lot of interest due to their linkage with early life catastrophic epileptic syndromes, such as infantile spasms, Ohtahara syndrome, X-linked myoclonic seizures, spasticity and intellectual disability, idiopathic infantile epileptic dyskinetic encephalopathy, X-linked mental retardation [63–66, 112–116] (reviewed in [117]). ARX is a transcription factor that regulates the proliferation and migration of GABA, calbindin, or neuropeptide Y positive interneurons but also of striatal cholinergic neurons [64, 66, 117]. Two recently published mouse models of ARX loss of function mutations, one of which specifically disrupted it in GABAergic interneurons destined to migrate to the neocortex, have recapitulated several phenotypes of infantile spasms and associated phenotype (cognitive, behavioral deficits and epileptogenesis) emphasizing the importance of deficient GABA inhibition for their pathogenesis [64, 66].

Angelman syndrome, a rare chromosomal deletion, involves the loss of ubiquitin-protein ligase 3A (UBE3A), but in certain patients there is a more extensive deletion of the 15q11-13 chromosomal locus that contains three GABA_A_ subunits, *α*5, *β*3, and *γ*3 GABA_A_ receptor subunits [118]. Genotype-phenotype correlation suggested that deletion of the GABA_A_ receptor subunits is associated with more severe seizures, including infantile spasms, atypical absences, and myoclonus whereas patients with UBE3A mutations had a milder phenotype [118]. The *β*3 subunit knockout mouse strain also develops a similar epilepsy phenotype [119].

Loss of function mutations of the voltage-sensitive sodium channel SCN1A gene is found in not only the severe myoclonic epilepsy of infancy (Dravet syndrome) but also in ADEFS^+^ syndrome [120–123]. SCN1A mutations have been proposed to preferentially impair the sodium channel activity of GABAergic interneurons, diminishing their activity [124]. Anti-NMDA autoantibodies detected in limbic encephalitis, a rare cause of refractory and frequent seizures [125], have been speculated to selectively target the NMDA receptors of presynaptic GABAergic terminals, reducing therefore GABA release [126].

Aberrant reappearance of depolarizing E_GABA_ and reduced GABA_A_ergic responses have been proposed to underlie the pathogenesis of seizures from cortical malformations. Pathology and electrophysiological studies from human tissue specimens from patients with cortical dysplasias, that commonly predispose to early life seizures, have also suggested the presence of depolarizing GABA [20, 127, 128]. In the neonatal freeze lesion model, a shift to the immature pattern of high NKCC1/KCC2 ratio in the lesional site [129] as well as reduced *γ*2 subunit expression and sensitivity to *α*1 subunit agonists in adulthood was described [130, 131]. In the rat model of cortical dysplasias induced by prenatal exposure to the 1-3-bis-chloroethyl-nitrosurea, reduced sensitivity to GABA was also seen in adulthood [132]. 

Traumatic brain injury in adults, such as in axotomized neurons, causes a reversal of GABA_A_ signaling and CCC expression profile to the immature pattern (more depolarizing GABA and dominant NKCC1 over KCC2 activity) [133–135]. This appears to aid the survival and regeneration process, promoting the brain-derived neurotrophic factor- (BDNF-) dependent neuronal survival and may resolve with time, during recovery [135]. However, there is limited information as to the consequences of neuronal trauma upon the expression, physiology, and connectivity of GABAergic interneurons in developing animals. In the partially isolated undercut cortical model, reduced GABA_A_ergic IPSCs and impaired chloride extrusion were found in juvenile rats, suggesting a possible correlation between impaired GABAergic inhibition and posttraumatic cortical excitability [136, 137]. Few studies have advocated against the use of GABA enhancing drugs and in favor of GABA_A_ receptor inhibitors as interventions to improve cognitive outcomes [138]. More detailed studies are needed to determine the role of posttraumatic GABA_A_ signaling changes for healing and regeneration in the developing brain as well as its impact on subsequent epileptogenesis and ensuing cognitive deficits.

## 7. Seizures Alter GABA_A_ Signaling

Seizures can affect almost every neurotransmitter system in the brain. Seizures can have immediate effects on GABA_A_ signaling, that is, during the ictal period, or delayed, appearing after the termination of seizures. In both scenarios, the observed changes are dynamic and evolving. Seizures may interfere with the expression, composition, and subcellular distribution of GABA_A_ receptors and their regulatory factors, such as CCCs or regulatory kinases. Defining the timing of these events is crucial, not only to better understand the pathophysiological mechanisms investigating these changes but also to best interpret their pathophysiological relevance for epileptogenesis and brain function. The temporal evolution of these events is also particularly important in developing rats, given the maturational changes that are ongoing. In addition, the age at first seizure, the type and severity of seizures, sex, epigenetic factors, medications, but also the cellular diversity of specific operant signaling systems further modify the final outcomes.

### 7.1. Ictal Attenuation of GABA_A_ Receptor-Mediated Inhibition

The urgency in treating early SE has long been recognized in the clinical literature. GABA-acting drugs, like benzodiazepines or barbiturates, are more effective early at onset of seizures than later on, when SE has been established [147, 148]. The transience of the efficacy of GABAergic drugs has been attributed to either increase internalization of selective synaptic GABA_A_ receptor subunits, such as of *β*2/3 and *γ*2, which mediate the effects of benzodiazepines and barbiturates [139, 140]. On the other hand, extrasynaptically located subunits that mediate tonic GABA inhibition, like the *δ* subunit, are not affected [139]. Failure of GABA_A_ receptor-mediated inhibition during prolonged seizures may also occur due to a positive shift in E_GABA_ either because of buildup of intracellular Cl^−^ concentration, from intense GABA_A_ receptor-mediated chloride inward pumping, or from impaired chloride extrusion mechanisms, due to increased NKCC1 activity or decreased KCC2-mediated Cl^−^ efflux [149–151].

### 7.2. Postictal Changes

Loss of GABAergic interneurons is a hallmark pathology of focal epilepsies, like mesial temporal sclerosis [152–157]. In experimental studies, prolonged seizures can lead to interneuronal loss but such effects are age-specific. In newborn rats, during the first week of life, even 3 episodes of status epilepticus (SE) do not injure GABAergic neurons [30]; yet cell death becomes a progressively more prominent feature as the age at exposure to SE increases [155, 158–160]. In contrast, early life seizures functionally disrupt the physiology of GABA_A_ receptor system. Age at the time of seizures, etiology or model of seizures, biological factors such as sex, as well as cell type and region-specific features may determine the end effects upon GABA_A_ receptor subunits or the direction of GABA_A_ receptor-mediated responses (Tables 3 and 4). These changes may be either compensatory attempts to repair or restore normal function or, on the contrary, may contribute to the postictal dysfunction, comorbidities, or sequelae of seizures, such as cognitive dysfunction or epileptogenesis. Unlike the adults, in which the physiology of GABA_A_ receptor-mediated signaling has reached a relative steady state, developmental research is further complicated by the evolving changes that normally occur during the period when brain matures[161]. There is no systematic research study taking us step-by-step through all the complexity of seizure-induced postictal alterations in GABA_A_ receptor physiology and any extrapolations should be cautiously done pending confirmation by actual experimentations. 

Seizures selectively interfere with the expression of certain, but not all, GABA_A_ receptor subunits [141–146] (Table 3). Kainic acid SE at PN9 rats favors the preservation of the immature pattern of GABA_A_ receptor complex (less *α*1, more *α2/α3* subunits) on the third postictal week [144] that typically attributes slower IPSC kinetics and less sensitivity to benzodiazepines. Similarly, recurrent flurothyl-induced seizures, in the first 10 days of life, decrease *α*1 expression and the amplitude of GABA_A_ receptor-mediated IPSCs [141–143]. Looking at longer-term outcomes of early life seizures, during adulthood, Brooks-Kayal's group has demonstrated that age at onset of SE is key at defining the final composition of GABA_A_ receptors and that this, in turn, may contribute to epileptogenesis. Lithium-pilocarpine SE at PN10 increases *α*1 subunit expression in the dentate granule cells in adulthood; in contrast, if SE is induced at PN20, a decrease in *α*1 subunit is noted, but only in the epileptic animals [145, 146]. Interestingly, reconstitution of *α*1 subunit expression prevented the occurrence of spontaneous seizures [146, 162].

The reports of untimely appearance of depolarizing GABA_A_ receptor signaling in a subpopulation of subicular neurons from adult human epileptic resected temporal lobes have attracted a lot of interest as a possible mechanism of epileptogenicity and potential refractoriness to GABA-acting antiepileptics [163, 164]. Depolarizing GABA_A_ receptor signaling has been linked to a dominance of NKCC1 over KCC2 activity in certain neurons of the epileptic tissue. It may also occur because of effective replenishment of intracellular bicarbonate by carbonic anhydrase during intense GABA_A_ receptor activation, which leads to a depolarization and to a consequent influx of Cl^−^, that enhances KCC2-mediated K^+^/Cl^−^ efflux [109]. The sequential interaction between carbonic anhydrase/GABA_A_ receptors/KCC2 may therefore increase extracellular K^+^, a factor that promotes the generation of ictal events. In support, carbonic anhydrase inhibitors have been used in certain cases as anticonvulsant therapies [109, 165].

 Seizures in adult animals tend to increase the ratio of NKCC1 over KCC2 activity, reverting to a more immature pattern of CCC balance that favors depolarizing E_GABA_ [151, 166]. This is believed to occur in humans as well [127, 167–170]. But what happens, then, after early life seizures, when neurons are already in an immature state and how does this impact epileptogenesis and functional outcomes? In the immediate postictal period, following brief recurrent kainic acid seizures or an hour of kainic acid SE, KCC2 is reshuffled towards the plasma membrane, increasing its capacity to export Cl^−^ [171]. As a result E_GABA_ becomes more negative, contributing perhaps to the ability of the neurons to stop seizures. 

In the longer run, further changes in E_GABA_ function occur, which are attributed to altered CCC expression or activity [30]. In our lab, we were interested in determining whether the original E_GABA_, at the time seizures occur, may control the effects of seizures on CCCs and the direction of GABA_A_ receptor-mediated signaling, in other words, whether seizures might have different effects upon GABA_A_ receptor-mediated signaling in neurons with depolarizing or hyperpolarizing GABA_A_ receptor mediated responses at the time of seizures. Taking advantage from the earlier appearance of GABA_A_ receptor currents in females than in males, we compared the effects of 3 episodes of kainic acid SE elicited at PN4, 5, and 6 (3KA-SE) in CA1 pyramidal neurons with depolarizing E_GABA_ (i.e., male) or isoelectric/hyperpolarizing E_GABA_ (i.e., female) at the time of seizures [30]. We found that 3KA-SE caused only a transient appearance of depolarizing GABA_A_ receptor mediated responses in neurons that had already started to shift to mature and more hyperpolarizing E_GABA_, similar to what was previously described for the adult neurons. In contrast, in male neurons, with still depolarizing GABAergic responses, 3KA-SE caused a precocious emergence of mature, hyperpolarizing responses. These changes were attributed to altered expression and/or activity of KCC2 and NKCC1. The precocious termination of depolarizing GABA_A_ signaling would be expected to deprive brain from its neurotrophic effects that are important for normal development [72, 74]. Indeed, 3KA-SE-exposed pups develop learning and memory problems when they grow up (unpublished data). However, the inability of the immature neurons to persistently exhibit depolarizing GABA_A_ receptor-mediated responses after seizures could be a protective feature against the development of subsequent epilepsy [30]. Our results indicate that age-specific factors, including the depolarizing GABA, may be important for this protection. Another dual regulator of CCCs and E_GABA_ through development is the brain-derived neurotrophic factor (BDNF) pathway, which is also activated in certain seizure models. BDNF increases KCC2 in developing neurons but decreases it in mature neurons [172, 173]. The opposite patterns of KCC2 regulation by BDNF in certain systems has been proposed to be due to trkB-mediated activation of different intracellular signaling cascades that regulate KCC2 expression [151].

The maturation of GABA_A_ receptor system occurs asynchronously across different neuronal types and brain regions. As a result, since early life seizures change the direction and strength of GABA_A_ receptor-mediated inhibition, their effects will be region and cell type specific, further confusing the interneuronal communication protocols. They may also disrupt the basic neural processes of learning and cognitive processing that depend upon GABA neurotransmission, such as long-term potentiation (LTP) [174–176], or social interactions [177–182]. The result will be a state of postictal confusion or more sustained cognitive or behavioral deficits [6]. Of interest, bumetanide treatment has shown benefit in five infants with autism [183]. However the exact mechanisms underlying this therapeutic effect are not yet known.

## 8. Implications for Early Life Seizures and Their Treatment

Human and experimental evidence indicates that similar to adults, aberrant preservation of depolarizing GABA_A_ signaling may also be a feature of the medically refractory epileptogenic focus in early life epilepsies. At present we do not have any data to discuss the pathological features of the medically sensitive early life epilepsies. The idea of pharmacologically enhancing GABA inhibition to stop seizures by using NKCC1 inhibitors like bumetanide is under investigation as a rationally developed, smart intervention to overcome the barriers posed by the well-established molecular switch of GABA_A_ receptor function [21]. Beneficial effects have been shown in few animal models [21, 186–189] and a human case report [190]. However, model-specific differences, as well as the timing of administration, can influence its efficacy in suppressing seizures [96, 191]. Moreover, concerns have been raised about potential adverse developmental effects on innocent bystander normal brain tissues, as may occur in chronic use in patients with focal epilepsies [74]. Undoubtedly, more studies need to be done to determine which seizure types are more likely to respond, when is the optimal time to administer, for how long, and how such interventions influence long-term outcomes in subjects who have already experienced seizures or have epilepsy. Similarly, by increasing our knowledge about the specific changes that occur in GABA_A_ receptor composition and pharmacology, it may be possible to design more selective and specific GABA_A_ receptor agonists for the very young or epileptic brain that is refractory to the existing medications. At the anatomical and electrophysiological level, it might be feasible, one day, to design such specific, very targeted, and individualized therapies to enhance GABA inhibition and stop seizures. The biggest challenge will be however to predict the functional state of GABA_A_ receptor-mediated inhibition at the target areas, so as to implement such rational therapies. Emerging evidence suggests that GABA-acting drugs, hormones, and different stressors are among the factors that can alter GABA_A_ receptor signaling, rendering it almost a moving target [11, 30, 31, 192–196]. The need for biomarkers of GABA_A_ function is therefore a priority.

## 9. Conclusion

The study of GABA in seizure generation and consequences has become a very fruitful field not only by generating intriguing results but also by producing challenging new questions. We have learned a number of mechanisms that compromise GABA_A_ inhibition in the very young or epileptic brain, predisposing to seizures and the associated cognitive and neurodevelopmental deficits. We still need to better understand and, most importantly, predict which is the normal balance between excitation and inhibition with sufficient age, sex, cell type, and regional, context, and function-related specificity, so as to preserve normal brain function and development.

## Figures and Tables

**Table 1:** GABA-related mutations linked with seizures.

GABA-related mutations	Species	Epilepsy type	Age at first observation	Ref.
GABA_A_ * receptor mutations*				

GABRA1	Human	ADJME, CAE	Childhood, Juvenile	[47, 48]
GABRA6	Human	CAE	Childhood	[49]
GABRB3	Human	CAE	Childhood	[50–52]
GABRD	Human	ADJME	Juvenile	[53]
GABRE	Human	Febrile, ADEFS^+^ IGE	Infantile, childhood	[49]
GABRG2	Human, mouse	CAE^+^ Febrile, ADEFS^+^, SMEI ADEFS^+^, SMEI, Febrile	Infantile, childhood	[54–59]
GABRP	Human	IGE, ADEFS^+^, Febrile	?	[49]

*Other mutations*				

GAD65 knockout	Mouse	Stress-induced, Limbic seizures	12 weeks	[60, 61]
ARX mutations	Human, mice	Early life epileptic encephalopathies (infantile spasms, Ohtahara)	Neonatal, Infantile	[62–66]

**Table 2:** Phenotype of CCC mutations.

CCC	Location	Mutation	Species	Neurological effect	Ref.
KCC1	Ubiquitous	Knockout	Mouse	None seen	[92]
KCC2	Brain	KCC2a and KCC2b knockout	Mouse	Death at birth	[93]
	Brain	KCC2b knockout	Mouse	Seizures, low weight, early mortality	[94]
	Brain	Hypomorph	Mouse	Increased seizure susceptibility and anxiety	[95]
	Brain	Heterozygote	Mouse	Hyperexcitability	[96]
KCC3	Ubiquitous	KCC3a-c knockout	Human, mouse	Peripheral neuropathy; seizures have been reported	[97–100]
KCC4	Kidney, heart, lungs, liver	Knockout	Mouse	Deafness	[101]
NKCC1	Ubiquitous	NKCC1a knockout	Mouse	Deafness, circling behavior	[102]
	Ubiquitous	NKCC1a and NKCC1b knockout	Mouse	Deafness, circling behavior, growth retardation, defective spermatogenesis, increased threshold to thermal stimulation	[103, 104]
NKCC2	Kidney	Knockout	Human	Bartter's syndrome	[105]

**Table 3:** Effects of early life seizures on *GABA*_*A*_ receptors and currents in rats.

Seizure model	Age	Region	Effects on GABA_A_ receptors	Ref.
*Ictal changes*				

In vivo SE (Lithium-pilocarpine; continuous hippocampal stimulation)	PN30	Hippocampus	Reduced surface expression of *β*2/3, *γ*2 subunits but not of *δ*.	[139]
In vivo SE (lithium-pilocarpine)	4–7 week old	Hippocampus	Internalization of *β*2/3, *γ*2 subunits; reduced mIPSCs	[140]

*After seizures*				

Recurrent flurothyl seizures	PN1-5	Hippocampus, somatosensory cortex	Decreased amplitude of GABAergic IPSCs	[141, 142]
Flurothyl seizures	PN6 or PN6-10	Hippocampus	Decreased numbers of *α*1-ir neurons	[143]
Kainic acid SE	PN9	Hippocampus	At 3 weeks postictally: *α*1, *α*4, *γ2* decrease; *α*2, *α*3 increase; *α*5 increase (CA3 only); *β3* increase compared to controls	[144]
Lithium-pilocarpine	PN10	Hippocampus (dentate gyrus)	In adulthood: increased *α*1 expression, larger GABA current, enhanced zolpidem sensitivity	[145]
Lithium-pilocarpine SE	PN20	Hippocampus	Decreased *α*1 and increased *α*4 expression in the hippocampus of epileptic versus non-epileptic rats	[146]

**Table 4:** Effects of Seizures on CCCs.

Model	Species	Age at seizures	Region	Effects	Ref.
*Ictal changes*					

Kainic acid	Rat	PN6-7	Hippocampus	Switch from hyperpolarizing to depolarizing E_GABA_	[184]
Low Mg^2+^ seizures	Mice	PN5	Hippocampus	Bumetanide sensitive increase in [Cl^−^]_i_	[150]

*After seizures*					

Kainic acid	Rat (male)	PN4-6	Hippocampus (at least 4 days postictally)	Increased KCC2; decreased NKCC1 activity; more hyperpolarizing E_GABA_	[185]
Kainic acid	Rat (female)	PN4-6	Hippocampus (at least 4 days postictally)	No change in KCC2; increased NKCC1 activity; more depolarizing E_GABA_	[185]
Kainic acid	Rat (male)	PN5-7	Hippocampus (immediate postictal period)	Increased surface expression of KCC2; hyperpolarizing shift of E_GABA_	[171]

## Abbreviations

ADEFS^+^: Autosomal dominant epilepsy with febrile seizures plus
ADJME: Autosomal dominant juvenile myoclonic epilepsy
ADNFLE: Autosomal dominant nocturnal frontal lobe epilepsy
ARX: Aristaless-related X-linked homeobox gene
BDNF: Brain-derived neurotrophic factor
CAE: Childhood absence epilepsy
GABA: Gamma aminobutyric acid
GABR: GABA_A_ receptor
GAD: Glutamic acid decarboxylase
IGE: Idiopathic generalized epilepsy
IPSC: Inhibitory postsynaptic current
3KA-SE: 3 episodes of kainic acid SE at PN4,5,6
KCC: Potassium chloride cotransporter
LTP: Long-term potentiation
NKCC: Sodium potassium chloride cotransporter
PN: Postnatal day
PTZ: Pentylenetetrazole
SCN1A: Sodium channel 1A
SMEI: Severe myoclonic epilepsy of infancy
SE: Status epilepticus
TLE: Temporal lobe epilepsy
UBE3A: Ubiquitin-protein ligase 3A.

## References

[1] Berg AT, Berkovic SF, Brodie MJ (2010). Revised terminology and concepts for organization of seizures and epilepsies: report of the ILAE Commission on Classification and Terminology, 2005–2009. *Epilepsia*.

[2] Fisher RS, van Emde Boas W, Blume W (2005). Epileptic seizures and epilepsy: definitions proposed by the International League Against Epilepsy (ILAE) and the International Bureau for Epilepsy (IBE). *Epilepsia*.

[3] Hauser WA, Annegers JF, Kurland LT (1993). Incidence of epilepsy and unprovoked seizures in Rochester, Minnesota: 1935–1984. *Epilepsia*.

[4] Kotsopoulos IAW, van Merode T, Kessels FGH, de Krom MCTFM, Knottnerus JA (2002). Systematic review and meta-analysis of incidence studies of epilepsy and unprovoked seizures. *Epilepsia*.

[5] Galanopoulou AS, Vidaurre J, Moshé SL (2002). Under what circumstances can seizures produce hippocampal injury: evidence for age-specific effects. *Developmental Neuroscience*.

[6] Pisani F, Cerminara C, Fusco C, Sisti L (2007). Neonatal status epilepticus vs recurrent neonatal seizures: clinical findings and outcome. *Neurology*.

[7] Padgett CL, Slesinger PA (2010). GABAB receptor coupling to G-proteins and ion channels. *Advances in Pharmacology*.

[8] McClellan KM, Calver AR, Tobet SA (2008). GABAB receptors role in cell migration and positioning within the ventromedial nucleus of the hypothalamus. *Neuroscience*.

[9] White JH, McIllhinney RAJ, Wise A (2000). The GABA_B_ receptor interacts directly with the related transcription factors CREB2 and ATFx. *Proceedings of the National Academy of Sciences of the United States of America*.

[10] Nehring RB, Horikawa HPM, El Far O (2000). The metabotropic GABA_B_ receptor directly interacts with the activating transcription factor 4. *Journal of Biological Chemistry*.

[11] Galanopoulou AS (2008). GABA_A_ receptors in normal development and seizures: friends or foes?. *Current Neuropharmacology*.

[12] Farrant M, Kaila K (2007). The cellular, molecular and ionic basis of GABAA receptor signalling. *Progress in Brain Research*.

[13] Olsen RW, Li GD (2010). GABA_A_ receptors as molecular targets of general anesthetics: identification of binding sites provides clues to allosteric modulation. *Canadian Journal of Anesthesia*.

[14] Blaesse P, Airaksinen MS, Rivera C, Kaila K (2009). Cation-chloride cotransporters and neuronal function. *Neuron*.

[15] Russell JM (2000). Sodium-potassium-chloride cotransport. *Physiological Reviews*.

[16] Plotkin MD, Snyder EY, Hebert SC, Delpire E (1997). Expression of the Na-K-2Cl cotransporter is developmentally regulated in postnatal rat brains: a possible mechanism underlying GABA’s excitatory role in immature brain. *Journal of Neurobiology*.

[17] Ben-Ari Y, Cherubini E, Corradetti R, Gaiarsa JL (1989). Giant synaptic potentials in immature rat CA3 hippocampal neurones. *Journal of Physiology*.

[18] Leinekugel X, Khalilov I, McLean H (1999). GABA is the principal fast-acting excitatory transmitter in the neonatal brain. *Advances in Neurology*.

[19] Rivera C, Voipio J, Payne JA (1999). The K^+^/Cl^−^ co-transporter KCC2 renders GABA hyperpolarizing during neuronal maturation. *Nature*.

[20] Jansen LA, Peugh LD, Roden WH, Ojemann JG (2010). Impaired maturation of cortical GABA_A_ receptor expression in pediatric epilepsy. *Epilepsia*.

[21] Dzhala VI, Talos DM, Sdrulla DA (2005). NKCC1 transporter facilitates seizures in the developing brain. *Nature Medicine*.

[22] Wang C, Shimizu-Okabe C, Watanabe K (2002). Developmental changes in KCC1, KCC2, and NKCC1 mRNA expressions in the rat brain. *Developmental Brain Research*.

[23] Stein V, Hermans-Borgmeyer I, Jentsch TJ, Hübner CA (2004). Expression of the KCl cotransporter KCC2 parallels neuronal maturation and the emergence of low intracellular chloride. *Journal of Comparative Neurology*.

[24] Kaila K, Voipio J, Paalasmaa P, Pasternack M, Deisz RA (1993). The role of bicarbonate in GABA_A_ receptor-mediated IPSPs of rat neocortical neurones. *Journal of Physiology*.

[25] Rivera C, Voipio J, Kaila K (2005). Two developmental switches in GABAergic signalling: the K^+^ − Cl^−^ cotransporter KCC2 and carbonic anhydrase CAVII. *Journal of Physiology*.

[26] Ruusuvuori E, Li H, Huttu K (2004). Carbonic anhydrase isoform VII acts as a molecular switch in the development of synchronous gamma-frequency firing of hippocampal CA1 pyramidal cells. *Journal of Neuroscience*.

[27] Scantlebury MH, Galanopoulou AS, Chudomelova L, Raffo E, Betancourth D, Moshé SL (2010). A model of symptomatic infantile spasms syndrome. *Neurobiology of Disease*.

[28] Vanhatalo S, Matias Palva J, Andersson S, Rivera C, Voipio J, Kaila K (2005). Slow endogenous activity transients and developmental expression of K^+^ − Cl^−^ cotransporter 2 in the immature human cortex. *European Journal of Neuroscience*.

[29] Galanopoulou AS (2008). Sexually dimorphic expression of KCC2 and GABA function. *Epilepsy Research*.

[30] Galanopoulou AS (2008). Dissociated gender-specific effects of recurrent seizures on GABA signaling in CA1 pyramidal neurons: role of GABAA receptors. *Journal of Neuroscience*.

[31] Galanopoulou AS, Kyrozis A, Claudio OI, Stanton PK, Moshé SL (2003). Sex-specific KCC2 expression and GABA_A_ receptor function in rat substantia nigra. *Experimental Neurology*.

[32] Nuñez JL, McCarthy MM (2007). Evidence for an extended duration of GABA-mediated excitation in the developing male versus female hippocampus. *Developmental Neurobiology*.

[33] Ben-Ari Y (2002). Excitatory actions of GABA during development: the nature of the nurture. *Nature Reviews Neuroscience*.

[34] Montenegro MA, Guerreiro MM, Caldas JPS, Moura-Ribeiro MVL, Guerreiro CAM (2001). Epileptic manifestations induced by midazolam in the neonatal period. *Arquivos de Neuro-Psiquiatria*.

[35] Connell J, Oozeer R, de Vries L, Dubowitz LMS, Dubowitz V (1989). Clinical and EEG response to anticonvulsants in neonatal seizures. *Archives of Disease in Childhood*.

[36] Booth D, Evans DJ (2004). Anticonvulsants for neonates with seizures. *Cochrane Database of Systematic Reviews*.

[37] Chiron C, Dulac O, Beaumont D, Palacios L, Pajot N, Mumford J (1991). Therapeutic trial of vigabatrin in refractory infantile spasms. *Journal of Child Neurology*.

[38] Lux AL, Edwards SW, Hancock E (2005). The United Kingdom Infantile Spasms Study (UKISS) comparing hormone treatment with vigabatrin on developmental and epilepsy outcomes to age 14 months: a multicentre randomised trial. *The Lancet Neurology*.

[39] Dzhala VI, Brumback AC, Staley KJ (2008). Bumetanide enhances phenobarbital efficacy in a neonatal seizure model. *Annals of Neurology*.

[40] Staley K (1992). Enhancement of the excitatory actions of GABA by barbiturates and benzodiazepines. *Neuroscience Letters*.

[41] Fritschy JM, Paysan J, Enna A, Mohler H (1994). Switch in the expression of rat GABA_A_-receptor subtypes during postnatal development: an immunohistochemical study. *Journal of Neuroscience*.

[42] Chudomel O, Herman H, Nair K, Moshé SL, Galanopoulou AS (2009). Age- and gender-related differences in GABAA receptor-mediated postsynaptic currents in GABAergic neurons of the substantia nigra reticulata in the rat. *Neuroscience*.

[43] Moshé SL, Sperber EF, Brown LL, Tempel A (1992). Age-dependent changes in substantia nigra GABA-mediated seizure suppression. *Epilepsy Research. Supplement*.

[44] Velikova J, Moshe SL (2001). Sexual dimorphism and developmental regulation of substantia nigra function. *Annals of Neurology*.

[45] Sperber EF, Velísková J, Germano IM, Friedman LK, Moshé SL (1999). Age-dependent vulnerability to seizures. *Advances in Neurology*.

[46] Kyrozis A, Chudomel O, Moshé SL, Galanopoulou AS (2006). Sex-dependent maturation of GABAA receptor-mediated synaptic events in rat substantia nigra reticulata. *Neuroscience Letters*.

[47] Cossette P, Liu L, Brisebois K (2002). Mutation of GABRA1 in an autosomal dominant form of juvenile myoclonic epilepsy. *Nature Genetics*.

[48] Ding L, Feng HJ, Macdonald RL, Botzolakis EJ, Hu N, Gallagher MJ (2010). GABA_A_ receptor *α*1 subunit mutation A322D associated with autosomal dominant juvenile myoclonic epilepsy reduces the expression and alters the composition of wild type GABA_A_ receptors. *Journal of Biological Chemistry*.

[49] Dibbens LM, Harkin LA, Richards M (2009). The role of neuronal GABA_A_ receptor subunit mutations in idiopathic generalized epilepsies. *Neuroscience Letters*.

[50] Urak L, Feucht M, Fathi N, Hornik K, Fuchs K (2006). A GABRB3 promoter haplotype associated with childhood absence epilepsy impairs transcriptional activity. *Human Molecular Genetics*.

[51] Feucht M, Fuchs K, Pichlbauer E (1999). Possible association between childhood absence epilepsy and the gene encoding GABRB3. *Biological Psychiatry*.

[52] Tanaka M, Olsen RW, Medina MT (2008). Hyperglycosylation and reduced GABA currents of mutated GABRB3 polypeptide in remitting childhood absence epilepsy. *American Journal of Human Genetics*.

[53] Dibbens LM, Feng HJ, Richards MC (2004). GABRD encoding a protein for extra- or peri-synaptic GABAA receptors is susceptibility locus for generalized epilepsies. *Human Molecular Genetics*.

[54] Baulac S, Huberfeld G, Gourfinkel-An I (2001). First genetic evidence of GABA_A_ receptor dysfunction in epilepsy: a mutation in the *γ*2-subunit gene. *Nature Genetics*.

[55] Sun H, Zhang Y, Liang J (2008). SCN1A, SCN1B, and GABRG2 gene mutation analysis in Chinese families with generalized epilepsy with febrile seizures plus. *Journal of Human Genetics*.

[56] Harkin LA, Bowser DN, Dibbens LM (2002). Truncation of the GABA_A_-receptor *γ*2 subunit in a family with generalized epilepsy with febrile seizures plus. *American Journal of Human Genetics*.

[57] Kang JQ, Shen W, Macdonald RL (2009). The GABRG2 mutation, Q351X, associated with generalized epilepsy with febrile seizures plus, has both loss of function and dominant-negative suppression. *Journal of Neuroscience*.

[58] Hirose S (2006). A new paradigm of channelopathy in epilepsy syndromes: intracellular trafficking abnormality of channel molecules. *Epilepsy Research*.

[59] Audenaert D, Schwartz E, Claeys KG (2006). A novel GABRG2 mutation associated with febrile seizures. *Neurology*.

[60] Kash SF, Johnson RS, Tecott LH (1997). Epilepsy in mice deficient in the 65-kDa isoform of glutamic acid decarboxylase. *Proceedings of the National Academy of Sciences of the United States of America*.

[61] Asada H, Kawamura Y, Maruyama K (1996). Mice lacking the 65 kDa isoform of glutamic acid decarboxylase (GAD65) maintain normal levels of GAD67 and GABA in their brains but are susceptible to seizures. *Biochemical and Biophysical Research Communications*.

[62] Kato M (2006). A new paradigm for West syndrome based on molecular and cell biology. *Epilepsy Research*.

[63] Kato M, Das S, Petras K (2004). Mutations of ARX are associated with striking pleiotropy and consistent genotype-phenotype correlation. *Human Mutation*.

[64] Marsh E, Fulp C, Gomez E (2009). Targeted loss of Arx results in a developmental epilepsy mouse model and recapitulates the human phenotype in heterozygous females. *Brain*.

[65] Kato M, Koyama N, Ohta M, Miura K, Hayasaka K (2010). Frameshift mutations of the ARX gene in familial Ohtahara syndrome. *Epilepsia*.

[66] Price MG, Yoo JW, Burgess DL (2009). A triplet repeat expansion genetic mouse model of infantile spasms syndrome, *Arx*
^(GCG)10+7^, with interneuronopathy, spasms in infancy, persistent seizures, and adult cognitive and behavioral impairment. *Journal of Neuroscience*.

[67] Frei MG, Zaveri HP, Arthurs S (2010). Controversies in epilepsy: debates held during the Fourth International Workshop on Seizure Prediction. *Epilepsy and Behavior*.

[68] Margineanu DG (2010). Epileptic hypersynchrony revisited. *NeuroReport*.

[69] Klaassen A, Glykys J, Maguire J, Labarca C, Mody I, Boulter J (2006). Seizures and enhanced cortical GABAergic inhibition in two mouse models of human autosomal dominant nocturnal frontal lobe epilepsy. *Proceedings of the National Academy of Sciences of the United States of America*.

[70] Mann EO, Mody I (2008). The multifaceted role of inhibition in epilepsy: seizure-genesis through excessive GABAergic inhibition in autosomal dominant nocturnal frontal lobe epilepsy. *Current Opinion in Neurology*.

[71] Danober L, Deransart C, Depaulis A, Vergnes M, Marescaux C (1998). Pathophysiological mechanisms of genetic absence epilepsy in the rat. *Progress in Neurobiology*.

[72] Cancedda L, Fiumelli H, Chen K, Poo MM (2007). Excitatory GABA action is essential for morphological maturation of cortical neurons in vivo. *Journal of Neuroscience*.

[73] Nakanishi K, Yamada J, Takayama C, Oohira A, Fukuda A (2007). NKCC1 activity modulates formation of functional inhibitory synapses in cultured neocortical neurons. *Synapse*.

[74] Wang DD, Kriegstein AR (2011). Blocking early GABA depolarization with bumetanide results in permanent alterations in cortical circuits and sensorimotor gating deficits. *Cerebral Cortex*.

[75] Maljevic S, Krampfl K, Cobilanschi J (2006). A mutation in the GABA_A_ receptor *α*1-subunit is associated with absence epilepsy. *Annals of Neurology*.

[76] Kang JQ, Shen W, Lee M, Gallagher MJ, Macdonald RL (2010). Slow degradation and aggregation in vitro of mutant GABAA receptor *γ*2(Q351X) subunits associated with epilepsy. *Journal of Neuroscience*.

[77] Galanopoulou AS (2010). Mutations affecting GABAergic signaling in seizures and epilepsy. *Pflügers Archiv European Journal of Physiology*.

[78] Macdonald RL, Kang JQ, Gallagher MJ (2010). Mutations in GABAA receptor subunits associated with genetic epilepsies. *Journal of Physiology*.

[79] Chiu C, Reid CA, Tan HO (2008). Developmental impact of a familial GABAA receptor epilepsy mutation. *Annals of Neurology*.

[80] Kaufman DL, Houser CR, Tobin AJ (1991). Two forms of the *γ*-aminobutyric acid synthetic enzyme glutamate decarboxylase have distinct intraneuronal distributions and cofactor interactions. *Journal of Neurochemistry*.

[81] Walls AB, Eyjolfsson EM, Smeland OB (2010). Knockout of GAD65 has major impact on synaptic GABA synthesized from astrocyte-derived glutamine. *Journal of Cerebral Blood Flow and Metabolism*.

[82] Walls AB, Nilsen LH, Eyjolfsson EM (2010). GAD65 is essential for synthesis of GABA destined for tonic inhibition regulating epileptiform activity. *Journal of Neurochemistry*.

[83] Kure S, Sakata Y, Miyahayashi S (1998). Mutation and polymorphic marker analyses of 65K- and 67K-glutamate decarboxylase genes in two families with pyridoxine-dependent epilepsy. *Journal of Human Genetics*.

[84] Gospe SM (2006). Pyridoxine-dependent seizures: new genetic and biochemical clues to help with diagnosis and treatment. *Current Opinion in Neurology*.

[85] Gospe SM, Olin KL, Keen CL (1994). Reduced GABA synthesis in pyridoxine-dependent seizures. *The Lancet*.

[86] Asada H, Kawamura Y, Maruyama K (1997). Cleft palate and decreased brain *γ*-aminobutyric acid in mice lacking the 67-kDa isoform of glutamic acid decarboxylase. *Proceedings of the National Academy of Sciences of the United States of America*.

[87] Ji F, Kanbara N, Obata K (1999). GABA and histogenesis in fetal and neonatal mouse brain lacking both the isoforms of glutamic acid decarboxylase. *Neuroscience Research*.

[88] Kwan P, Sills GJ, Kelly K, Butler E, Brodie MJ (2000). Glutamic acid decarboxylase autoantibodies in controlled and uncontrolled epilepsy: a pilot study. *Epilepsy Research*.

[89] Yoshimoto T, Doi M, Fukai N (2005). Type 1 diabetes mellitus and drug-resistant epilepsy: presence of high titer of anti-glutamic acid decarboxylase autoantibodies in serum and cerebrospinal fluid. *Internal Medicine*.

[90] Pearce DA, Atkinson M, Tagle DA (2004). Glutamic acid decarboxylase autoimmunity in Batten disease and other disorders. *Neurology*.

[91] McKnight K, Jiang Y, Hart Y (2005). Serum antibodies in epilepsy and seizure-associated disorders. *Neurology*.

[92] Rust MB, Alper SL, Rudhard Y (2007). Disruption of erythroid K-Cl cotransporters alters erythrocyte volume and partially rescues erythrocyte dehydration in SAD mice. *Journal of Clinical Investigation*.

[93] Hübner CA, Stein V, Hermans-Borgmeyer I, Meyer T, Ballanyi K, Jentsch TJ (2001). Disruption of KCC2 reveals an essential role of K-Cl cotransport already in early synaptic inhibition. *Neuron*.

[94] Woo NS, Lu J, England R (2002). Hyperexcitability and epilepsy associated with disruption of the mouse neuronal-specific K-Cl cotransporter gene. *Hippocampus*.

[95] Tornberg J, Voikar V, Savilahti H, Rauvala H, Airaksinen MS (2005). Behavioural phenotypes of hypomorphic KCC2-deficient mice. *European Journal of Neuroscience*.

[96] Zhu L, Polley N, Mathews GC, Delpire E (2008). NKCC1 and KCC2 prevent hyperexcitability in the mouse hippocampus. *Epilepsy Research*.

[97] Dupré N, Howard HC, Mathieu J (2003). Hereditary motor and sensory neuropathy with agenesis of the corpus callosum. *Annals of Neurology*.

[98] Mathieu J, Bedard F, Prevost C, Langevin P (1990). Hereditary motor and sensory neuropathy with or without agenesis of the corpus callosum. Radiological and clinical study of 64 cases. *Canadian Journal of Neurological Sciences*.

[99] Howard HC, Mount DB, Rochefort D (2002). The K-Cl cotransporter KCC3 is mutant in a severe peripheral neuropathy associated with agenesis of the corpus callosum. *Nature Genetics*.

[100] Boettger T, Rust MB, Maier H (2003). Loss of K-Cl co-transporter KCC3 causes deafness, neurodegeneration and reduced seizure threshold. *The EMBO Journal*.

[101] Boettger T, Hübner CA, Maier H, Rust MB, Beck FX, Jentsch TJ (2002). Deafness and renal tubular acidosis in mice lacking the K-Cl co-transporter Kcc4. *Nature*.

[102] Dixon MJ, Gazzard J, Chaudhry SS, Sampson N, Schulte BA, Steel KP (1999). Mutation of the Na-K-Cl co-transporter gene Slc12a2 results in deafness in mice. *Human Molecular Genetics*.

[103] Pace AJ, Madden VJ, Henson OW, Koller BH, Henson MM (2001). Ultrastructure of the inner ear of NKCC1-deficient mice. *Hearing Research*.

[104] Delpire E, Lu J, England R, Dull C, Thorne T (1999). Deafness and imbalance associated with inactivation of the secretory Na-K-2Cl co-transporter. *Nature Genetics*.

[105] Simon DB, Karet FE, Hamdan JM, Di Pietro A, Sanjad SA, Lifton RP (1996). Bartter’s syndrome, hypokalaemic alkalosis with hypercalciuria, is caused by mutations in the Na-K-2CI cotransporter NKCC2. *Nature Genetics*.

[106] Uvarov P, Ludwig A, Markkanen M (2007). A novel N-terminal isoform of the neuron-specific K-Cl cotransporter KCC2. *Journal of Biological Chemistry*.

[107] Staley KJ, Mody I (1992). Shunting of excitatory input to dentate gyrus granule cells by a depolarizing GABA_A_ receptor-mediated postsynaptic conductance. *Journal of Neurophysiology*.

[108] Li H, Khirug S, Cai C (2007). KCC2 interacts with the dendritic cytoskeleton to promote spine development. *Neuron*.

[109] Viitanen T, Ruusuvuori E, Kaila K, Voipio J (2010). The K^+^-Cl^−^ cotransporter KCC2 promotes GABAergic excitation in the mature rat hippocampus. *Journal of Physiology*.

[110] Wang DD, Kriegstein AR (2008). GABA regulates excitatory synapse formation in the neocortex via NMDA receptor activation. *Journal of Neuroscience*.

[111] Berg AT, Smith SN, Frobish D (2005). Special education needs of children with newly diagnosed epilepsy. *Developmental Medicine and Child Neurology*.

[112] Kitamura K, Yanazawa M, Sugiyama N (2002). Mutation of ARX causes abnormal development of forebrain and testes in mice and X-linked lissencephaly with abnormal genitalia in humans. *Nature Genetics*.

[113] Friocourt G, Poirier K, Rakić S, Parnavelas JG, Chelly J (2006). The role of ARX in cortical development. *European Journal of Neuroscience*.

[114] Strømme P, Mangelsdorf ME, Scheffer IE, Gécz J (2002). Infantile spasms, dystonia, and other X-linked phenotypes caused by mutations in Aristaless related homeobox gene, ARX. *Brain and Development*.

[115] Turner G, Partington M, Kerr B, Mangelsdorf M, Gecz J (2002). Variable expression of mental retardation, autism, seizures, and dystonic hand movements in two families with an identical ARX gene mutation. *American Journal of Medical Genetics*.

[116] Partington MW, Turner G, Boyle J, Gécz J (2004). Three new families with X-linked mental retardation caused by the 428-451dup(24bp) mutation in ARX. *Clinical Genetics*.

[117] Friocourt G, Parnavelas JG (2010). Mutations in ARX result in several defects involving GABAergic neurons. *Frontiers in Cellular Neuroscience*.

[118] Minassian BA, DeLorey TM, Olsen RW (1998). Angelman syndrome: correlations between epilepsy phenotypes and genotypes. *Annals of Neurology*.

[119] Homanics GE, DeLorey TM, Firestone LL (1997). Mice devoid of *γ*-aminobutyrate type A receptor *β*3 subunit have epilepsy, cleft palate, and hypersensitive behavior. *Proceedings of the National Academy of Sciences of the United States of America*.

[120] Berkovic SF, Harkin L, McMahon JM (2006). De-novo mutations of the sodium channel gene SCN1A in alleged vaccine encephalopathy: a retrospective study. *The Lancet Neurology*.

[121] Harkin LA, McMahon JM, Iona X (2007). The spectrum of SCN1A-related infantile epileptic encephalopathies. *Brain*.

[122] Heron SE, Scheffer IE, Iona X (2010). De novo SCN1A mutations in Dravet syndrome and related epileptic encephalopathies are largely of paternal origin. *Journal of Medical Genetics*.

[123] Wallace RH, Hodgson BL, Grinton BE (2003). Sodium channel *α*1-subunit mutations in severe myoclonic epilepsy of infancy and infantile spasms. *Neurology*.

[124] Martin MS, Dutt K, Papale LA (2010). Altered function of the SCN1A voltage-gated sodium channel leads to *γ*-aminobutyric acid-ergic (GABAergic) interneuron abnormalities. *Journal of Biological Chemistry*.

[125] Prüss H, Dalmau J, Harms L (2010). Retrospective analysis of NMDA receptor antibodies in encephalitis of unknown origin. *Neurology*.

[126] Iizuka T, Sakai F (2008). Anti-NMDA receptor encephalitis—clinical manifestations and pathophysiology. *Brain and Nerve*.

[127] Aronica E, Boer K, Redeker S (2007). Differential expression patterns of chloride transporters, Na^+^-K^+^-2Cl^−^-cotransporter and K^+^ − Cl^−^-cotransporter, in epilepsy-associated malformations of cortical development. *Neuroscience*.

[128] Cepeda C, André VM, Wu N (2007). Immature neurons and GABA networks may contribute to epileptogenesis in pediatric cortical dysplasia. *Epilepsia*.

[129] Shimizu-Okabe C, Okabe A, Kilb W, Sato K, Luhmann HJ, Fukuda A (2007). Changes in the expression of cation-Cl^−^ cotransporters, NKCC1 and KCC2, during cortical malformation induced by neonatal freeze-lesion. *Neuroscience Research*.

[130] Peters O, Redecker C, Hagemann G, Bruehl C, Luhmann HJ, Witte OW (2004). Impaired synaptic plasticity in the surround of perinatally aquired dysplasia in rat cerebral cortex. *Cerebral Cortex*.

[131] Hablitz JJ, DeFazio RA (2000). Altered receptor subunit expression in rat neocortical malformations. *Epilepsia*.

[132] Benardete EA, Kriegstein AR (2002). Increased excitability and decreased sensitivity to GABA in an animal model of dysplastic cortex. *Epilepsia*.

[133] Nabekura J, Ueno T, Okabe A (2002). Reduction of KCC2 expression and GABAA receptor-mediated excitation after in vivo axonal injury. *Journal of Neuroscience*.

[134] Toyoda H, Ohno K, Yamada J (2003). Induction of NMDA and GABAA receptor-mediated Ca^2+^ oscillations with KCC2 mRNA downregulation in injured facial motoneurons. *Journal of Neurophysiology*.

[135] Shulga A, Thomas-Crusells J, Sigl T (2008). Posttraumatic GABA_A_-mediated [Ca^2+^]_i_ increase is essential for the induction of brain-derived neurotrophic factor-dependent survival of mature central neurons. *Journal of Neuroscience*.

[136] Jin X, Huguenard JR, Prince DA (2005). Impaired Cl^−^ extrusion in layer V pyramidal neurons of chronically injured epileptogenic neocortex. *Journal of Neurophysiology*.

[137] Jin X, Huguenard JR, Prince DA (2011). Reorganization of inhibitory synaptic circuits in rodent chronically injured epileptogenic neocortex. *Cerebral Cortex*.

[138] Ochalski PG, Fellows-Mayle W, Hsieh LB (2010). Flumazenil administration attenuates cognitive impairment in immature rats after controlled cortical impact. *Journal of Neurotrauma*.

[139] Goodkin HP, Joshi S, Mtchedlishvili Z, Brar J, Kapur J (2008). Subunit-specific trafficking of GABA_A_ receptors during status epilepticus. *Journal of Neuroscience*.

[140] Naylor DE, Liu H, Wasterlain CG (2005). Trafficking of GABA_A_ receptors, loss of inhibition, and a mechanism for pharmacoresistance in status epilepticus. *Journal of Neuroscience*.

[141] Isaeva E, Isaev D, Khazipov R, Holmes GL (2006). Selective impairment of GABAergic synaptic transmission in the flurothyl model of neonatal seizures. *European Journal of Neuroscience*.

[142] Isaeva E, Isaev D, Khazipov R, Holmes GL (2009). Long-term suppression of GABAergic activity by neonatal seizures in rat somatosensory cortex. *Epilepsy Research*.

[143] Ni H, Jiang YW, Bo T, Wang JM, Wu XR (2005). c-Fos, N-methyl-D-aspartate receptor 2C, GABA-A-*α*1 immonoreactivity, seizure latency and neuronal injury following single or recurrent neonatal seizures in hippocampus of Wistar rat. *Neuroscience Letters*.

[144] Laurén HB, Lopez-Picon FR, Korpi ER, Holopainen IE (2005). Kainic acid-induced status epilepticus alters GABAA receptor subunit mRNA and protein expression in the developing rat hippocampus. *Journal of Neurochemistry*.

[145] Zhang G, Raol YH, Hsu FC, Coulter DA, Brooks-Kayal AR (2004). Effects of status epilepticus on hippocampal GABAA receptors are age-dependent. *Neuroscience*.

[146] Raol YH, Zhang G, Lund IV, Porter BE, Maronski MA, Brooks-Kayal AR (2006). Increased GABA_A_-receptor *α*1-subunit expression in hippocampal dentate gyrus after early-life status epilepticus. *Epilepsia*.

[147] Abend NS, Gutierrez-Colina AM, Dlugos DJ (2010). Medical treatment of pediatric status epilepticus. *Seminars in Pediatric Neurology*.

[148] Shearer P, Riviello J (2011). Generalized convulsive status epilepticus in adults and children: treatment guidelines and protocols. *Emergency Medicine Clinics of North America*.

[149] Lux HD, Heinemann U (1978). Ionic changes during experimentally induced seizure activity. *Electroencephalography and Clinical Neurophysiology. Supplement*.

[150] Dzhala VI, Kuchibhotla KV, Glykys JC (2010). Progressive NKCC1-dependent neuronal chloride accumulation during neonatal seizures. *Journal of Neuroscience*.

[151] Rivera C, Voipio J, Thomas-Crusells J (2004). Mechanism of activity-dependent downregulation of the neuron-specific K-Cl cotransporter KCC2. *Journal of Neuroscience*.

[152] Sayin U, Osting S, Hagen J, Rutecki P, Sutula T (2003). Spontaneous seizures and loss of axo-axonic and axo-somatic inhibition induced by repeated brief seizures in kindled rats. *Journal of Neuroscience*.

[153] Obenaus A, Esclapez M, Houser CR (1993). Loss of glutamate decarboxylase mRNA-containing neurons in the rat dentate gyrus following pilocarpine-induced seizures. *Journal of Neuroscience*.

[154] Wang L, Liu YH, Huang YG, Chen LW (2008). Time-course of neuronal death in the mouse pilocarpine model of chronic epilepsy using Fluoro-Jade C staining. *Brain Research*.

[155] Leite JP, Babb TL, Pretorius JK, Kuhlman PA, Yeoman KM, Mathern GW (1996). Neuron loss, mossy fiber sprouting, and interictal spikes after intrahippocampal kainate in developing rats. *Epilepsy Research*.

[156] Sloviter RS, Zappone CA, Harvey BD, Bumanglag AV, Bender RA, Frotscher M (2003). “Dormant basket cell” hypothesis revisited: relative vulnerabilities of dentate gyrus mossy cells and inhibitory interneurons after hippocampal status epilepticus in the rat. *Journal of Comparative Neurology*.

[157] Fritschy JM, Kiener T, Bouilleret V, Loup F (1999). GABAergic neurons and GABA_A_-receptors in temporal lobe epilepsy. *Neurochemistry International*.

[158] Haas KZ, Sperber EF, Opanashuk LA, Stanton PK, Moshé SL (2001). Resistance of immature hippocampus to morphologic and physiologic alterations following status epilepticus or kindling. *Hippocampus*.

[159] Stafstrom CE, Thompson JL, Holmes GL (1992). Kainic acid seizures in the developing brain: status epilepticus and spontaneous recurrent seizures. *Developmental Brain Research*.

[160] Nitecka L, Tremblay E, Charton G (1984). Maturation of kainic acid seizure-brain damage syndrome in the rat. II. Histopathological sequelae. *Neuroscience*.

[161] Laurie DJ, Wisden W, Seeburg PH (1992). The distribution of thirteen GABA_A_ receptor subunit mRNAs in the rat brain. III. Embryonic and postnatal development. *Journal of Neuroscience*.

[162] Raol YH, Lund IV, Bandyopadhyay S (2006). Enhancing GABA_A_ receptor *α*1 subunit levels in hippocampal dentate gyrus inhibits epilepsy development in an animal model of temporal lobe epilepsy. *Journal of Neuroscience*.

[163] Cohen I, Navarro V, Clemenceau S, Baulac M, Miles R (2002). On the origin of interictal activity in human temporal lobe epilepsy in vitro. *Science*.

[164] Huberfeld G, Wittner L, Clemenceau S (2007). Perturbed chloride homeostasis and GABAergic signaling in human temporal lobe epilepsy. *Journal of Neuroscience*.

[165] Reiss WG, Oles KS (1996). Acetazolamide in the treatment of seizures. *Annals of Pharmacotherapy*.

[166] Li X, Zhou J, Chen Z, Chen S, Zhu F, Zhou L (2008). Long-term expressional changes of Na^+^-K^+^ − Cl^−^ co-transporter 1 (NKCC1) and K^+^ − Cl^−^ co-transporter 2 (KCC2) in CA1 region of hippocampus following lithium-pilocarpine induced status epilepticus (PISE). *Brain Research*.

[167] Muñoz A, Méndez P, Defelipe J, Alvarez-Leefmans FJ (2007). Cation-chloride cotransporters and GABA-ergic innervation in the human epileptic hippocampus. *Epilepsia*.

[168] Palma E, Amici M, Sobrero F (2006). Anomalous levels of Cl^−^ transporters in the hippocampal subiculum from temporal lobe epilepsy patients make GABA excitatory. *Proceedings of the National Academy of Sciences of the United States of America*.

[169] Sen A, Martinian L, Nikolic M, Walker MC, Thom M, Sisodiya SM (2007). Increased NKCC1 expression in refractory human epilepsy. *Epilepsy Research*.

[170] Munakata M, Watanabe M, Otsuki T (2007). Altered distribution of KCC2 in cortical dysplasia in patients with intractable epilepsy. *Epilepsia*.

[171] Khirug S, Ahmad F, Puskarjov M, Afzalov R, Kaila K, Blaesse P (2010). A single seizure episode leads to rapid functional activation of KCC2 in the neonatal rat hippocampus. *Journal of Neuroscience*.

[172] Aguado F, Carmona MA, Pozas E (2003). BDNF regulates spontaneous correlated activity at early developmental stages by increasing synaptogenesis and expression of the K^+^/Cl^−^ co-transporter KCC2. *Development*.

[173] Rivera C, Li H, Thomas-Crusells J (2002). BDNF-induced TrkB activation down-regulates the K^+^ − Cl^−^ cotransporter KCC2 and impairs neuronal Cl^−^ extrusion. *Journal of Cell Biology*.

[174] Wang W, Gong N, Xu T-L (2006). Downregulation of KCC2 following LTP contributes to EPSP-spike potentiation in rat hippocampus. *Biochemical and Biophysical Research Communications*.

[175] Wang W, Wang H, Gong N, Xu TL (2006). Changes of K^+^ − Cl^−^ cotransporter 2 (KCC2) and circuit activity in propofol-induced impairment of long-term potentiation in rat hippocampal slices. *Brain Research Bulletin*.

[176] McBain CJ, Kauer JA (2009). Presynaptic plasticity: targeted control of inhibitory networks. *Current Opinion in Neurobiology*.

[177] Chao HT, Chen H, Samaco RC (2010). Dysfunction in GABA signalling mediates autism-like stereotypies and Rett syndrome phenotypes. *Nature*.

[178] Di Cristo G (2007). Development of cortical GABAergic circuits and its implications for neurodevelopmental disorders. *Clinical Genetics*.

[179] Collins AL, Ma D, Whitehead PL (2006). Investigation of autism and GABA receptor subunit genes in multiple ethnic groups. *Neurogenetics*.

[180] van Kooten IAJ, Hof PR, van Engeland H, Steinbusch HWM, Patterson PH, Schmitz C (2005). Autism: neuropathology, alterations of the GABAergic system, and animal models. *International Review of Neurobiology*.

[181] D’Hulst C, Heulens I, Brouwer JR (2009). Expression of the GABAergic system in animal models for fragile X syndrome and fragile X associated tremor/ataxia syndrome (FXTAS). *Brain Research*.

[182] DeLorey TM, Olsen RW (1999). GABA and epileptogenesis: comparing gabrb3 gene-deficient mice with Angelman syndrome in man. *Epilepsy Research*.

[183] Lemonnier E, Ben-Ari Y (2010). The diuretic bumetanide decreases autistic behaviour in five infants treated during 3 months with no side effects. *Acta Paediatrica*.

[184] Khalilov I, Holmes GL, Ben-Ari Y (2003). In vitro formation of a secondary epileptogenic mirror focus by interhippocampal propagation of seizures. *Nature Neuroscience*.

[185] Galanopoulou AS (2007). Developmental patterns in the regulation of chloride homeostasis and GABA_A_ receptor signaling by seizures. *Epilepsia*.

[186] Nardou R, Ben-Ari Y, Khalilov I (2009). Bumetanide, an NKCC1 antagonist, does not prevent formation of epileptogenic focus but blocks epileptic focus seizures in immature rat hippocampus. *Journal of Neurophysiology*.

[187] Mares P (2009). Age-and dose-specific anticonvulsant action of bumetanide in immature rats. *Physiological Research*.

[188] Brandt C, Nozadze M, Heuchert N, Rattka M, Löscher W (2010). Disease-modifying effects of phenobarbital and the NKCC1 inhibitor bumetanide in the pilocarpine model of temporal lobe epilepsy. *Journal of Neuroscience*.

[189] Mazarati A, Shin D, Sankar R (2009). Bumetanide inhibits rapid kindling in neonatal rats. *Epilepsia*.

[190] Kahle KT, Barnett SM, Sassower KC, Staley KJ (2009). Decreased seizure activity in a human neonate treated with bumetanide, an inhibitor of the Na^+^-K^+^-2Cl^−^ cotransporter NKCC1. *Journal of Child Neurology*.

[191] Kilb W, Sinning A, Luhmann HJ (2007). Model-specific effects of bumetanide on epileptiform activity in the in-vitro intact hippocampus of the newborn mouse. *Neuropharmacology*.

[192] Galanopoulou AS, Moshé SL (2003). Role of sex hormones in the sexually dimorphic expression of KCC2 in rat substantia nigra. *Experimental Neurology*.

[193] Hsu FC, Zhang GJ, Raol YSH, Valentino RJ, Coulter DA, Brooks-Kayal AR (2003). Repeated neonatal handling with maternal separation permanently alters hippocampal GABAA receptors and behavioral stress responses. *Proceedings of the National Academy of Sciences of the United States of America*.

[194] Ganguly K, Schinder AF, Wong ST, Poo MM (2001). GABA itself promotes the developmental switch of neuronal GABAergic responses from excitation to inhibition. *Cell*.

[195] Ceanga M, Spataru A, Zagrean AM (2010). Oxytocin is neuroprotective against oxygen-glucose deprivation and reoxygenation in immature hippocampal cultures. *Neuroscience Letters*.

[196] Khazipov R, Tyzio R, Ben-Ari Y (2008). Effects of oxytocin on GABA signalling in the foetal brain during delivery. *Progress in Brain Research*.

